# Intra-abdominal hypertension due to heparin - induced retroperitoneal hematoma in patients with ventricle assist devices: report of four cases and review of the literature

**DOI:** 10.1186/1749-8090-5-108

**Published:** 2010-11-10

**Authors:** Stavros I Daliakopoulos, Manja Schaedel, Michael N Klimatsidas, Sotirios Spiliopoulos, Reiner Koerfer, Gero Tenderich

**Affiliations:** 1 Herzzentrum Essen, Herwarthstrasse 100, 45138 Essen, Germany; 2 424 Military Hospital of Thessaloniki, Thoracic Surgery Department, 56429 Thessaloniki, Greece

## Abstract

**Introduction:**

Elevated intra-abdominal pressure (IAP) has been identified as a cascade of pathophysiologic changes leading in end-organ failure due to decreasing compliance of the abdomen and the development of abdomen compartment syndrome (ACS). Spontaneous retroperitoneal hematoma (SRH) is a rare clinical entity seen almost exclusively in association with anticoagulation states, coagulopathies and hemodialysis; that may cause ACS among patients in the intensive care unit (ICU) and if treated inappropriately represents a high mortality rate.

**Case Presentation:**

We report four patients (a 36-year-old Caucasian female, a 59-year-old White-Asian male, a 64-year-old Caucasian female and a 61-year-old Caucasian female) that developed an intra-abdominal hypertension due to heparin-induced retroperitoneal hematomas after implantation of ventricular assist devices because of heart failure. Three of the patients presented with dyspnea at rest, fatigue, pleura effusions in chest XR and increased heart rate although b-blocker therapy. A 36-year old female (the forth patient) presented with sudden, severe shortness of breath at rest, 10 days after an "acute bronchitis". At the time of the event in all cases international normalized ratio (INR) was <3.5 and partial thromboplastin time <65 sec. The patients were treated surgically, the large hematomas were evacuated and the systemic manifestations of the syndrome were reversed.

**Conclusion:**

Identifying patients in the ICU at risk for developing ACS with constant surveillance can lead to prevention. ACS is the natural progression of pressure-induced end-organ changes and develops if IAP is not recognized and treated in a timely manner. Failure to recognize and appropriately treat ACS is fatal while timely intervention - if indicated - is associated with improvements in organ function and patient survival. Means for surgical decision making are based on clinical indicators of adverse physiology, rather than on a single measured parameter.

## Background

Ventricular assist devices (VADs) have been demonstrated to be effective in either bridging patients with end-stage heart failure to transplantation or as long-term support - destination therapy - or as a bridge to myocardial recovery resulting in substantial improvement in survival rates [1,2]. For every 1000 patients with end-stage heart failure, the implantation of a left ventricular assist device could prevent at least 270 deaths annually. The treatment effect is nearly four times that of beta-blockers or angiotensin-converting - enzyme inhibitors (ACE inhibitors), which have been estimated to prevent 70 deaths for every 1000 patients treated who receive either type of agent [3,4]. The Achilles' heel of Prolonged Ventricular Assist Device Support has been right ventricular dysfunction and device-related complications, such as thromboembolism, infection, and bleeding. The latter is triggered by changes in the coagulation system [5,6] and remains the most common postoperative complication after VAD implantation, necessitating reoperation in up to 60% of cases irrespective of device used or indication for insertion.

Spontaneous retroperitoneal hematoma (SRH) on the other hand is a distinctive clinical entity, most commonly seen in association with patients with anticoagulation therapy, bleeding abnormalities, and haemodialysis [7,8] and may represent one of the most serious and potentially lethal complications of anticoagulation therapy [9]. The large study of Sasson et al. [10] showed that patients receiving heparin as anticoagulation therapy should be carefully monitored for the development of groin pain or leg weakness because of a SRH. Monica Mourthe et al reported the only case where abdominal compartment syndrome was related to this clinical entity [11].

The World Society of Abdominal Compartment Syndrome has defined Intra-abdominal hypertension as a sustained or repeated pathologic elevation of IAP ≥ 12 mmHg whereas the same society defined the Abdominal Compartment Syndrome as a sustained IAP > 20 mmHg associated with new organ dysfunction or failure, with signs of end-organ compromise, confirmed by alleviation of symptoms on abdominal decompression. Both of these entities compress the pulmonary parenchyma which results in an increased intrapulmonary shunt fraction.

## 1^st ^Case presentation

The 1^st ^case we report is of a 36-year-old Caucasian female with severe heart failure secondary to virus induced myocarditis that required biventricular support with Thoratec PVAD^(r) ^ventricular assist device (Thoratec Laboratories Corp, Pleasanton, CA). She was initially treated with Furosemid (Lasix^(r)^) 500 mg/50 ml NaCl with a rate of 5-10 mg/h, ACE inhibitors, and dobutamin^(r) ^250 mg/50 ml with a rate of 10 μg/KG BW/min. Despite maximal medical treatment, including levosimendan (Simdax^(r)^) 25 mg/500 ml G5% with a rate of 0.1 μg/KG BW/min, her clinical and hemodynamic status deteriorated 36 hours later with hypotension, cardiac index (CI) of 1.60 L/min/m^2 ^and cardiogenic shock, with threatening multiple organ failure. The patient was evaluated and accepted for ventricular assist device implantation.

Postoperatively, after spending 128 hours in the ICU and while in mechanical ventilation, her liver and kidney function promptly recovered, the inotropic agents were reduced, and the patient remained clinically stable under dobutamin & dopamine and heparin IV. Heparin therapy was monitored three times per day, using the partial thromboplastin time (aPTT) and the dose was adjusted to attain the target 50 - 60 sec.

**Figure 1:**
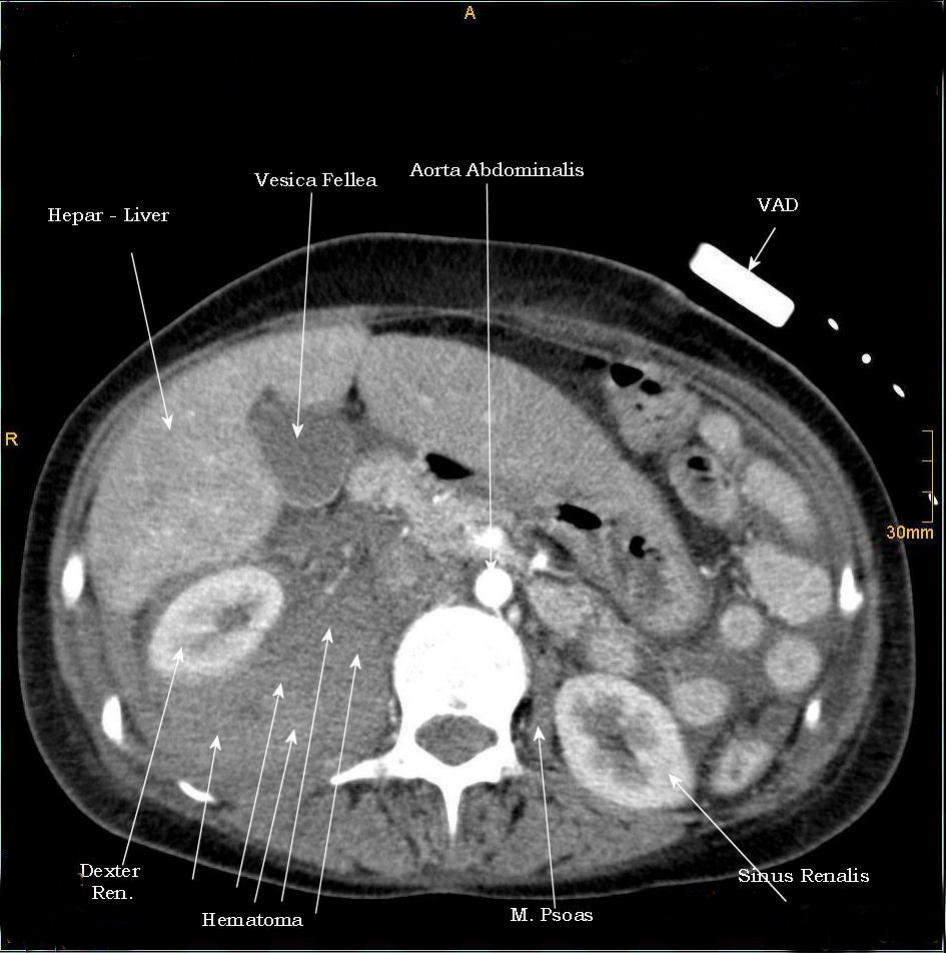
**1^st ^case**. CT - axial plan demonstrating a retroperitoneal hematoma adherent to the right psoas muscle, shifting the right renal lateral.

On the 7^th ^ICU-day the patient developed a tense, distended abdomen and became oliguric. Pulmonary vascular resistance was 305 dyn × sec/cm^5^. Abdominal ultrasound revealed an empty bladder with a urinary catheter in situ and kidneys of normal size. Despite to an adequate mean arterial pressure (65 mm Hg) and passage of a nasogastric tube to decompress the stomach, oliguria persisted. Intraabdominal pressure (IAP) was measured via a urinary catheter and was shown to be 27 mm Hg, which confirmed abdominal compartment syndrome (ACS) [12]. CT of the abdomen and pelvis showed a large retroperitoneal hematoma (Figure 1). The patient was initially treated with transfusion of 8 units of packed red cells (PRC) and 4 units of fresh frozen plasma (FFP). Despite adequate fluid and blood product resuscitation the patient remained unstable so that the large retroperitoneal hematoma had to be surgically removed on the 8^th ^ICU-day. The patient remained in the ICU for 47 days.

## 2^nd ^Case presentation

**Figure 2:**
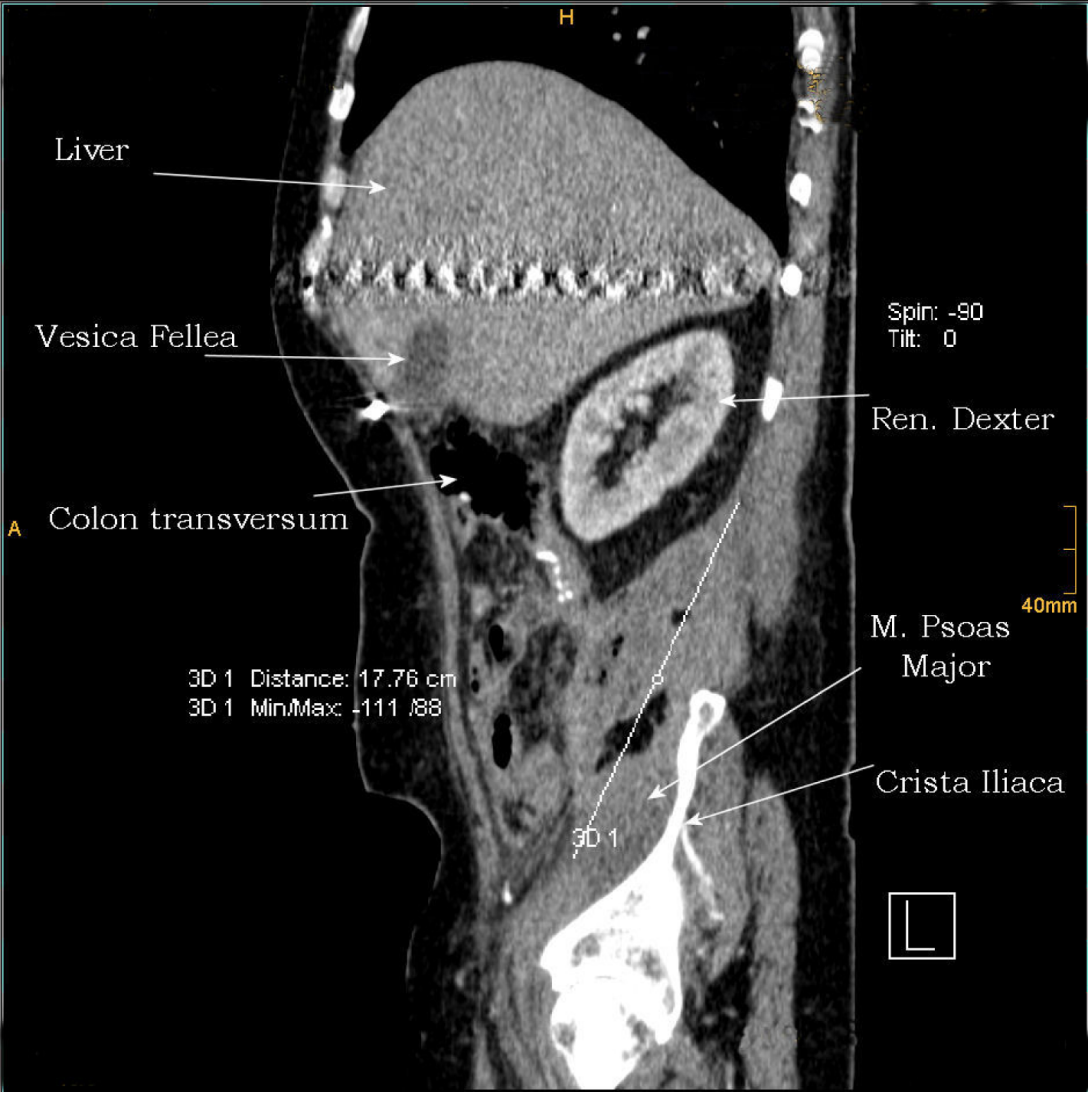
**2^nd ^case**. CT - sagittal plan of a large retroperitoneal hematoma - 17.76 cm.

**Figure 3:**
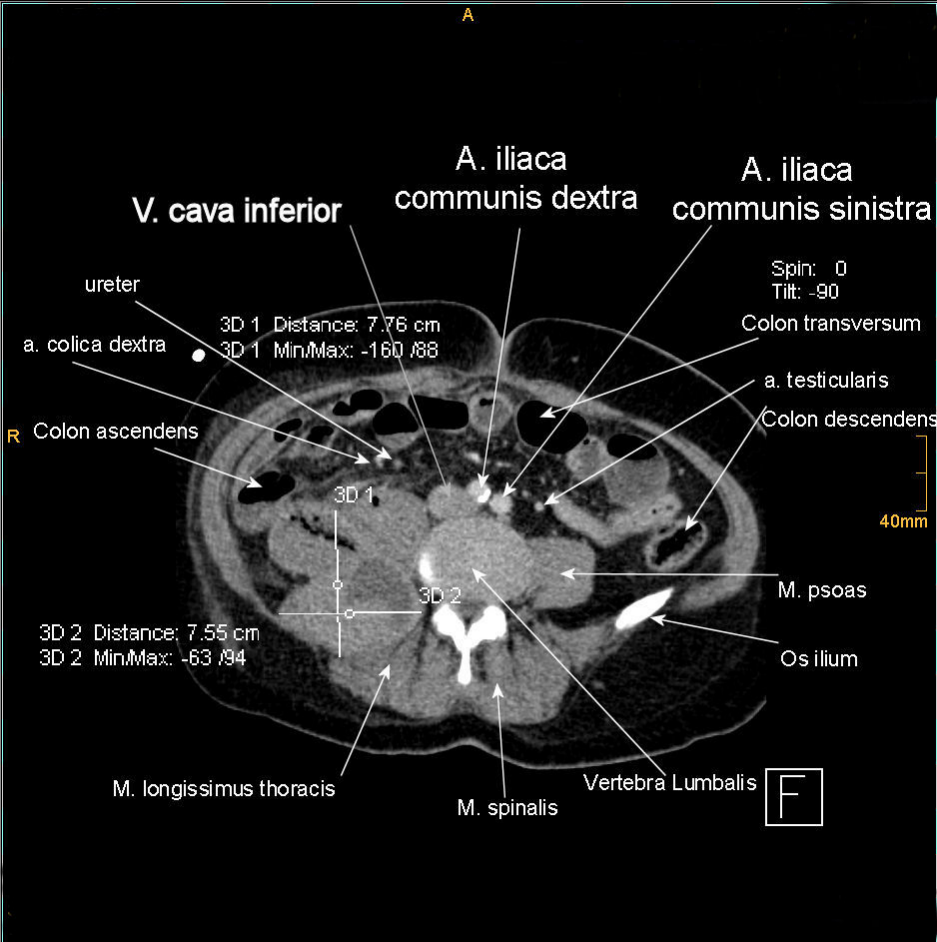
**2^nd ^case**. CT - axial plan of the hematoma shifting the right ureter to the middle line.

A 59-year-old White-Asian male was admitted to hospital and required support with Heart Mate II Thoratec^(r) ^LVAS because of terminal heart insufficient due to idiopathic dilated cardiomyopathy. On the 6^th ^ICU-day hemodynamic indicators included elevated heart rate (HF > 140 b/min), hypotension (Systolic/Diastolic BP 60/40 mm Hg), elevated Pulmonary Artery Wedge Pressure (27 mmHg) and Central Venous Pressure (CVP 16 mmHg) with elevated Systemic - SVR: 1500 dyn × sec/cm^5 ^and Pulmonary - PVR: 345 dyn × sec/cm^5 ^Vascular Resistance made the patient's mechanical ventilation difficult, requiring high peak inflating pressures (P_max _34 mmHg and high positive expiratory end-pressure (PEEP > 10) in order to maintain adequate oxygenation. During the next hours the patient became anuric with IAP of 22 mmHg. CT revealed a 17,76 cm (Figure 2, 3) retroperitoneal hematoma that was surgically removed. The retroperitoneum had to be packed and a re-exploration was necessary 72 h later before the final closure. The patient was discharged from the ICU on 56^th ^postoperative day (after LVAD implantation).

## 3^rd ^Case presentation

**Figure 4:**
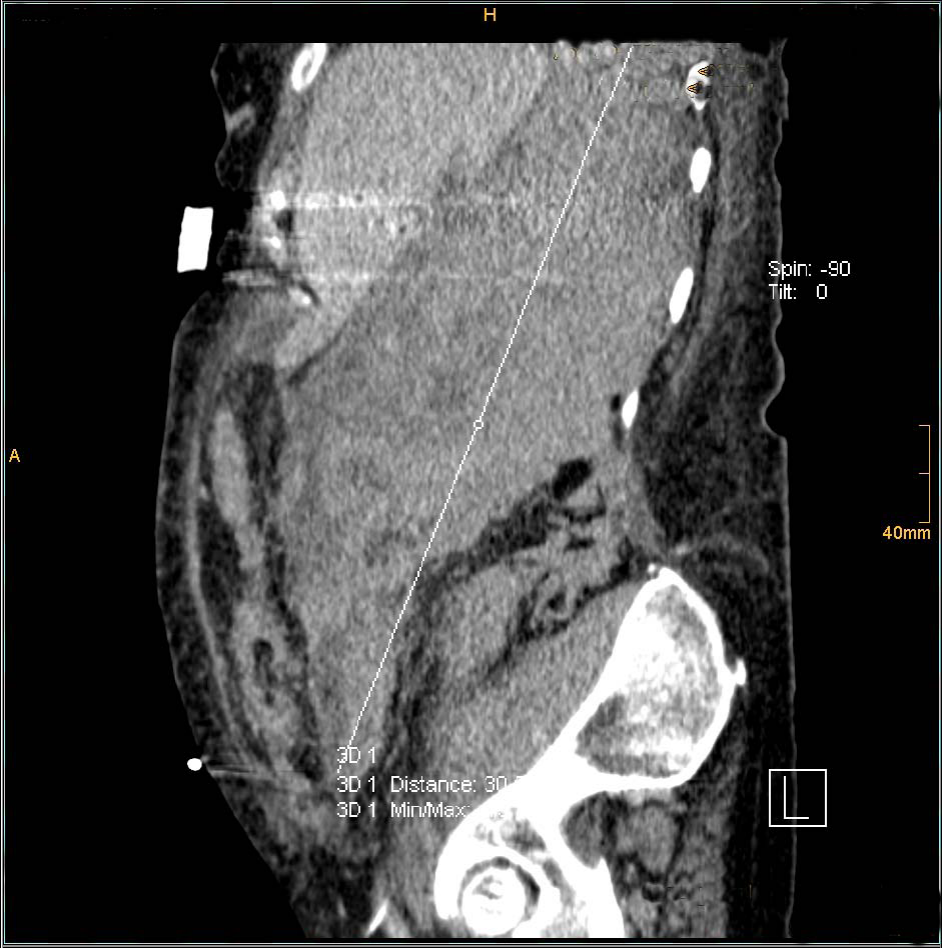
**3^rd ^case**. CT - sagittal plan demonstrating a 30 cm hematoma.

**Figure 5:**
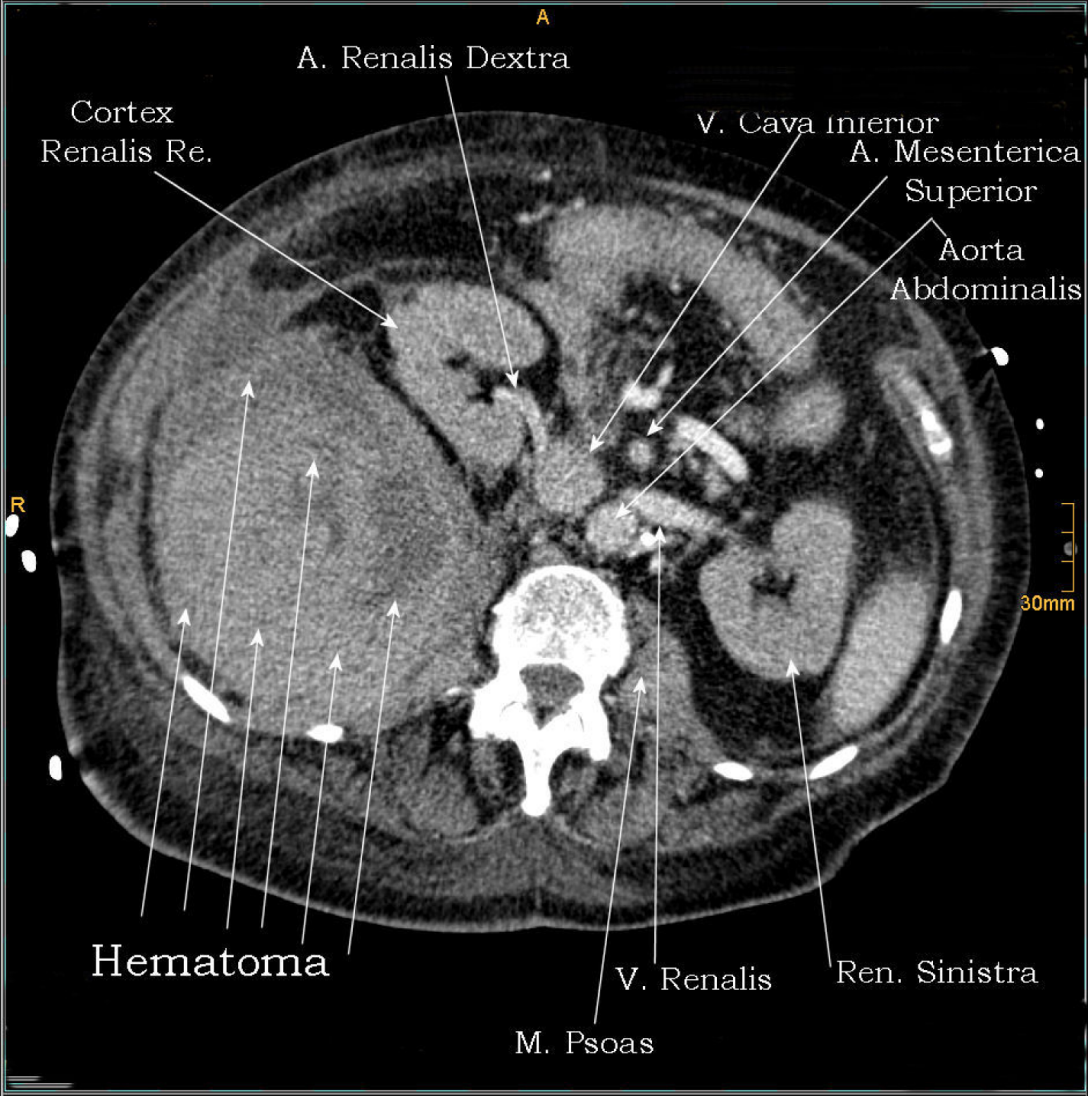
**3^rd ^case**. CT - axial plan of a huge hematoma shifting the whole right renal to the middle line.

**Figure 6:**
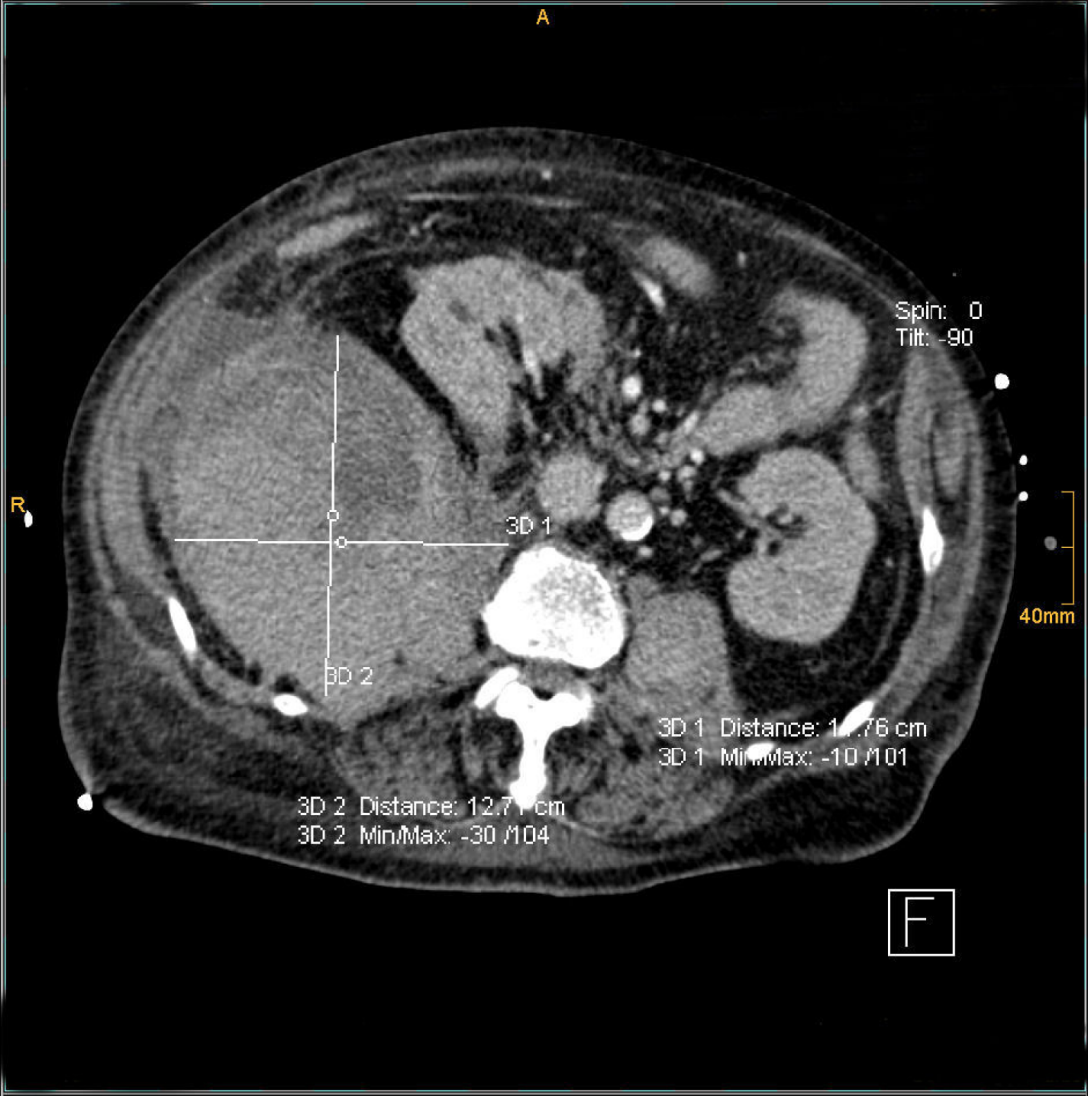
**3^rd ^case**. CT - axial plan of the hematoma.

A 64-year-old Caucasian female on 10^th ^postoperative day after Heart Mate II Thoratec^(r) ^LVAS became anuric while IAP was 23 mmHg. CT revealed a 30 cm retroperitoneal hematoma that was surgically removed (Figure 4, 5, 6). The patient died on the 89^th ^postoperative day in the ICU because of multiple organs insufficiency.

## 4^th ^Case presentation

**Figure 7:**
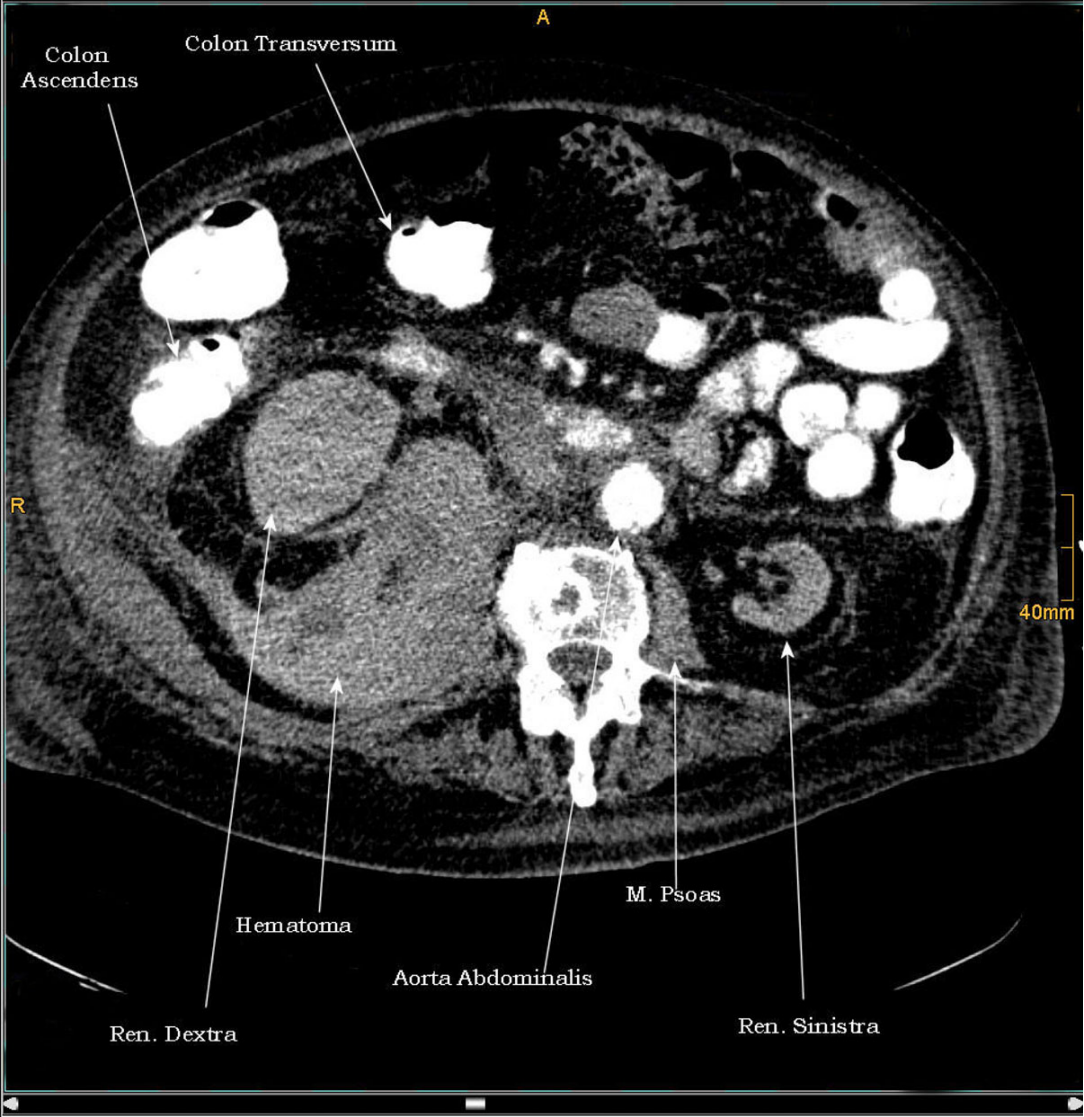
**4^th ^case**. CT - axial plan of the hematoma.

A 61-year-old Caucasian female required mechanical ventilation and dialysis due to respiratory distress syndrome and anuria on 13^th ^postoperative day after Heart Mate II Thoratec^(r) ^LVAS. CT on 15^th ^postoperative day revealed a large retroperitoneal hematoma that was surgically removed (Figure 7). The patient remained in the ICU for 63 days.

## Discussion

Postoperative hemorrhage is common among patients with VADs and many of them have risk factors predisposing to hemorrhage. Risk factors for significant hemorrhage include coagulopathy due to hepatic congestion associated with severe heart failure, compromised nutritional status, preoperative anticoagulation therapies, and previous cardiac surgery [13]. Although extensive bleeding usually occurs into the mediastinum or pericardial space, VADs can have other complications not confined to the chest. Hemolysis and resulting biliary complications are common and according to John R. [14] and Kamdar F. [15] axial flow devices (Heart Mate II to our cases) seem to be associated with higher rate of gastrointestinal bleeding, ventricular arrhythmias and intracranial hemorrhage.

All of our patients developed IAH as a consequence of large retroperitoneal hematoma and reduced intra-abdominal volume. This was inferred by changes in the patient's hepatic transaminases and was manifested by oliguria, raised abdominal pressure and inadequate oxygenation result in hypercapnia and acidosis requiring high PEEP and peak ventilator pressures, which exacerbate the hemodynamic abnormalities.

Retroperitoneal hematoma among patients in the ICU is a well-recognized but relative rare condition with an incidence of 0.1%, although has been reported at 0.6 - 6.6% of patients undergoing therapeutic anticoagulation [16,17]. Warfarin, unfractioned and low-molecular heparin have all been implicated [18,9].

All the patients in our cases before operation and in order to receive a LAD or a Bi-VAD they were examined for Heparin Induced Thrombocytopenia (ELISA & HIPAA). In all cases the HIT test was negative. After the implantation of the assist device the number of platelets was reduced but the post- operation labor examination didn't provide any signs of HIT.

Appendix 1 demonstrates the 4 Grades of IAH according to the World Society of the Abdominal Compartment Syndrome. The mortality rate in patients with IAH and ACS varies from 29 to 62% and is usually due to multiple organ failure and sepsis [19-21]. A diverse range of associated conditions may lead to from IAH to ACS requiring aggressive fluid resuscitation (Appendix 2).

The earliest manifestation of ACS is reported by Eddy et al. [22] to be the pulmonary dysfunction. IAP is transmitted to the thorax both directly and through cephalad deviation of the diaphragm. This significantly increases intrathoracic pressure resulting in extrinsic compression of the pulmonary parenchyma and development of pulmonary dysfunction [23,24]. Increased intrapleural pressures resulting from transmitted intra-abdominal forces produce elevations in measured hemodynamic parameters including CVP and PAWP resulting in false LVAD or PVAD settings. In our series of cases we noted that accurate prediction of end-diastolic filling pressures was no longer reliable to be made from PAWP equations but via transoesophageal echocardiography. Significant hemodynamic changes have been demonstrated with IAP above 20 mmHg [25].

Oliguria or even anuria develops despite measured normal or mildly elevated CPV and PAWP due to IAH-induced reductions in renal blood flow and function [26,27]. Because of IAP renal vein and renal vascular resistance are both significant elevated leading to impaired glomerular and tubular renal function and reduction in urinary output [23,26,27]. Nevertheless interesting is the fact that renal failure in the absence of pulmonary dysfunction is not likely to be the result of IAH [22].

Because many of the effects of ACS are clinically indistinguishable from those of other common entities related to critically ill patients, it is probable that the influence of an elevated IAP is not infrequently missed in a patient with multifactorial complications. As a result, clinicians must possess a high index of suspicion and monitor IAP frequently. Contemporary measurement of the IAP outside of the laboratory is accomplished by a variety of means. These include direct measurement of IAP by means of an intra-peritoneal catheter, as is done during laparoscopy. Bedside measurement of IAP has been accomplished by transduction of pressures from indwelling femoral vein, rectal, gastric and urinary bladder catheters. The latter method is used in our institution and is possible by measuring intra-cystic pressure (ICP) as a reflection of IAP using a Foley catheter [28-30] although large series of human studies correlating ICP and IAP are lacking to date [31]. Continuous Intra-cystic pressure measure was used to determine the IAP indirectly at the era of the first signs of IAH.

Chest radiography can be used to evaluate gross positioning of the pump and the inflow and outflow cannulas or may show elevated hemidiaphragms with loss of lung volume but these findings seem to be difficult to identify in patients with VADs. These changes have been demonstrated with IAP above 15 mmHg [25]. Transoesophageal echocardiography was routinely employed to all of our patients during the intraoperative and perioperative periods to evaluate thrombus formation, pump flow, mechanical complications and ventricular filling and uploading but CT detected in all cases the problem. Common CT features included extrinsic compression of the inferior vena cava (IVC), positive round belly sign and an anteroposterior-to-transverse abdominal ratio of more than 80 [32].

The usual treatment of ACS by decompression of the abdomen, often by laparotomy, in those with moderately elevated intra-abdominal pressure is growing in vogue [12,33], although conservative treatment is comprised of supportive therapy and abdominal decompression with nasogastric tube and flatus tube.

In our cases the indication of open surgery ACS was complicated of the presence of the large retroperitoneal hematoma. We didn't proceed to a decompressive laparotomy because all of the hematomas were so tense that the possibility of anterior eruption after abdominal pressure released was high. We preferred to remove the large hematoma in order to avoid this phenomenon and in one case we had to pack and re-explore the retroperitoneum because of diffuse bleeding.

Before operating hematological values were restored and coagulopathy cascade was corrected by replacement of coagulation factors. In all patients from the second postoperative day (after LVAD or PVAD implantation) and till weaning from mechanical ventilation (MV) unfractioned heparin was used in continuous 24 h pump perfusion without discharge aiming a target aPTT 50-60 sec. After weaning from MV and two days after the last drainage was removed all of the patients received additional anticoagulation therapy, initially phenprocoumon 3 mg (Marcumar^(r)^) aiming a target INR 2.5-3.5 and finally acetylsalicylsäure (ASS^(r) ^100 mg/day). Marcumar^(r) ^and ASS^(r) ^were not discontinued after hospital discharge.

To avoid a reperfusion syndrome from the release of acid and metabolites from reperfused tissues after the abdomen decompression [34,35]. we used in all cases a two liter solution consisting of 0.45% Normal Saline with 50 gr of Mannitol and 50 mEq of Sodium Bicarbonate [36].

## Conclusion

IAH has a significant role in contributing to the early multiple organ dysfunction syndrome (MODS). The presentation is varied and may be vague and diagnosis is often delayed. The patients who have retroperitoneal hematoma as cause of the IAH often do not have any obvious clinical signs. Relative hypotension and mild tachycardia are most of the time present. Any abnormal and sudden increase in the volume of any component of the intra-peritoneal or extra-peritoneal spaces can cause Intra-abdominal Hypertension. When associated with organ dysfunction (elevated airway pressure, cardiac output reduction and oliguria) it meets the criteria for Abdomen Compartment Syndrome. Treatment consists of prompt surgical decompression, volemic resuscitation and any further strategy is based on recognition of resultant organ dysfunction.

Our report finally indicates that ACS can occur outside the typical setting of abdominal surgery or trauma, decompressive laparotomy is not always the gold standard and patients with VADs may be at high risk for postoperative IAH and ACS.

## Competing interests

The authors declare that they have no competing interests.

## Consent

Written informed consent was obtained from our patients for publication of this case report and any accompanying images. A copy of the written consent is available for review by the Editor-in-Chief of this journal.

## Authors' contributions

SID participated in the sequence alignment, designing the case report and drafting the manuscript. MS participated in the design of the case report. MNK participated in the design of the case report. SS participated in the design and culled relevant information. RK participated in the design of the case report. GT coordinated the preparation of the case report and designed the whole manuscript. All authors read and approved the final manuscript.

## Appendices

### Appendix 1

IAH Grading System_according to the WSACS_

Grade I: IAP 12-15 mmHg

Grade II: IAP 16-20 mmHg

Grade III: IAP 21-25 mmHg

Grade IV: IAP > 25 mmHg

### Appendix 2

Risk factors responsible for IAH/ACS_according to the WSACS_

Mechanical ventilation

Acidosis (pH < 7,2)

Polytransfusion (>10U Packed Red Blood/24 h)

Hypothermia (core temperature <33°C)

Sepsis

Bacteremia

Intra-abdominal infection/abscess

Pneumonia

Peritoneal Dialysis

Abdominal surgery, especially with fascial closures

Massive fluid resuscitation (>5 lt colloid or crystalloid/24 h)

Gastroparesis - gastric distention - ileus

Major burns

Major trauma

Prone positioning

Massive incisional hernia repair

Damage control laparotomy

Laparoscopy with excessive inflation pressures

High Body Mass Index (>30 Kg/m^2^)

Coagulopathy

Liver dysfunction/cirrhosis with ascites

Hemoperitoneum/pneumoperitoneum

Acute pancreatitis

Peritonitis

Intra-abdominal or retroperitoneal tumors
